# Comparing blends and blocks: Synthesis of partially fluorinated diblock polythiophene copolymers to investigate the thermal stability of optical and morphological properties

**DOI:** 10.3762/bjoc.12.205

**Published:** 2016-10-10

**Authors:** Pierre Boufflet, Sebastian Wood, Jessica Wade, Zhuping Fei, Ji-Seon Kim, Martin Heeney

**Affiliations:** 1 Department of Chemistry and Centre for Plastic Electronics, Imperial College London, Exhibition Rd, London, SW7 2AZ, UK; 2 Department of Physics and Centre for Plastic Electronics, Imperial College London, London, SW7 2AZ, UK

**Keywords:** conjugated block-copolymer synthesis, fluorination, microphase stabilization, polythiophene, temperature-dependent Raman spectroscopy

## Abstract

The microstructure of the active blend layer has been shown to be a critically important factor in the performance of organic solar devices. Block copolymers provide a potentially interesting avenue for controlling this active layer microstructure in solar cell blends. Here we explore the impact of backbone fluorination in block copolymers of poly(3-octyl-4-fluorothiophene)s and poly(3-octylthiophene) (F-P3OT-b-P3OT). Two block co-polymers with varying block lengths were prepared via sequential monomer addition under Kumada catalyst transfer polymerisation (KCTP) conditions. We compare the behavior of the block copolymer to that of the corresponding homopolymer blends. In both types of system, we find the fluorinated segments tend to dominate the UV–visible absorption and molecular vibrational spectral features, as well as the thermal behavior. In the block copolymer case, non-fluorinated segments appear to slightly frustrate the aggregation of the more fluorinated block. However, in situ temperature dependent Raman spectroscopy shows that the intramolecular order is more thermally stable in the block copolymer than in the corresponding blend, suggesting that such materials may be interesting for enhanced thermal stability of organic photovoltaic active layers based on similar systems.

## Introduction

With thin-film microstructure playing such a key role in the optoelectronic and charge transport properties of conjugated polymers, block copolymers naturally appear as useful tools for tailoring the thin-film morphology [1–5]. The propensity of some block copolymers to phase segregate at the nano-scale is of particular interest in the field of organic photovoltaics (OPV), where separation of the electron donor and acceptor domains on the order of the exciton diffusion length (5–10 nm) is required [1,6–9]. Many approaches have been reported to tether two or more light-absorbing polymers to form a block copolymer [1,10–15], with some attempts focusing on one block being the donor and the other the acceptor [16–18]. The main objective is that the blocks spontaneously “phase-separate“ on the necessary length scales for efficient charge separation, transport and collection. With the thermal stability of all-polymer and polymer-fullerene blend microstructures being particularly problematic [19], block copolymers present a potential solution. Indeed, it has been shown that when block copolymers are used as additives in bulk heterojunction donor–acceptor blend layers, the morphology of the resulting ternary blend film can be more stable over long periods of time, even under thermal annealing [14,20–21].

However, the synthesis of block copolymers can be difficult to control, particularly in the case of step-growth polymerizations that are often used to synthesize conjugated polymers. In these polymerizations, such as Stille or Suzuki polycondensations, one approach to the block copolymer synthesis is the addition of a well-defined polymeric end-capper, commonly bromine terminated poly(3-hexylthiophene). An issue with this approach can be the formation of a mixture of di- and triblock copolymers, which adds to the complexity of the system and makes batch-to-batch reproducibility difficult [1–2]. Particularly problematic is the lack of control over the block lengths and molecular weight for the step-growth polymerization. Indeed, the relative block lengths play a key role in the morphology control and the self-assembly behavior of these polymers [7,22–23]. The Grignard Metathesis (GRIM) polymerization, also known as the Kumada catalyst transfer polymerisation (KCTP), is a popular method to synthesize conjugated block copolymers because its chain growth behavior avoids any issues of triblock copolymers, and also provides good control over the molecular weight and relative block lengths [24–31]. In addition to enabling the formation of a diblock copolymer via sequential monomer addition*,* the KCTP can lead to controlled end-functionalization [1,28,32]. This has been used as a handle for further applications such as macroinitiation [7,10,21,31,33], endcapping [34], and grafting [35–39]. Despite the KCTP having somewhat limited scope and functional group tolerance, its advantages in terms of synthetic control mean that it is one of the most common methods for synthesizing fully conjugated block copolymers.

Since the backbone flexibility of each block has a crucial impact on the self-assembly of a block copolymer [7,40], the properties of polythiophene-based block copolymers can potentially be tuned by backbone fluorination which increases backbone rigidity [41–42]. As an initial exploration, this contribution presents the synthesis and purification of two block copolymers of poly(3-octylthiophene) (P3OT) and poly(3-fluoro-4-octylthiophene) (F-P3OT) with different relative block lengths. The thermal behavior of the polymers’ UV–visible absorption and Raman scattering spectra are compared with those of the corresponding blends of P3OT and F-P3OT, as well as the homopolymers. The results of this study suggest that the tethering of P3OT and F-P3OT blocks may not lead to spontaneous large-scale phase separation behavior, but critically increases the thermal stability of intramolecular order, as observed by temperature dependent Raman spectroscopy studies.

## Results and Discussion

### Synthesis

**Scheme 1:**
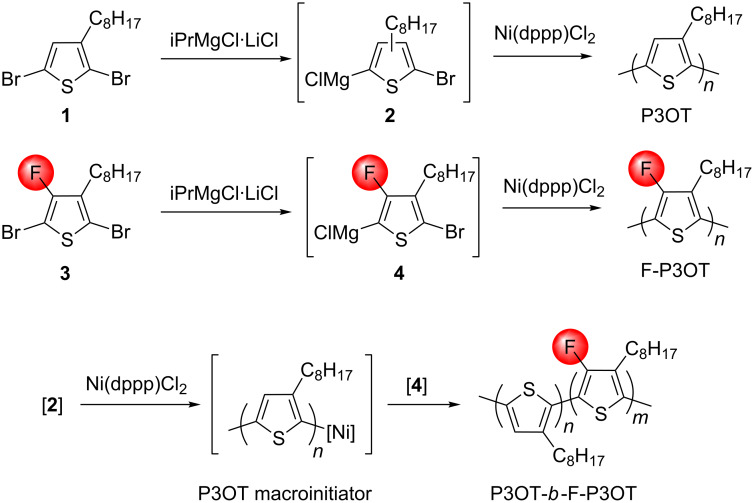
Grignard metathesis polymerization method of synthesizing the diblock copolymer. Relative block lengths are modified by changing the feed ratio of **2** to **4**.

The monomers and homopolymers P3OT and F-P3OT were synthesized via KCTP from the activated monomers **2** and **4**, as reported in our previous work (Scheme 1) [42]. The precipitated polymers were purified by Soxhlet extraction, washing sequentially with methanol, acetone and hexane (and chloroform for F-P3OT). P3OT was then extracted using chloroform, and F-P3OT with chlorobenzene.

The P3OT-*b*-F-P3OT copolymers were also synthesized by KCTP using a method analogous to that used for thiophene-selenophene block copolymers [26]. Due to the much lower solubility of F-P3OT compared to P3OT, the more soluble P3OT block was grown first from the activated monomer **2**, followed by the addition of **4** to the P3OT macroinitiator. Relative block lengths were controlled by varying the relative feed ratios of **2** to **4**. In order to probe the effect of block lengths on the polymer properties, 1:3 and 3:1 feed ratios of **2** to **4** were used, respectively. True block-lengths as determined by ^1^H NMR (vide infra) were found to be 1:4 and 2:1, and the polymers will be referred to as such hereafter.

The block copolymers were initially purified by Soxhlet extraction, washing sequentially with methanol, acetone and hexane. In order to remove any P3OT homopolymer that may have been produced through chain termination prior to the addition of **4**, the P3OT-*b*-F-P3OT polymers were washed with a solvent that could selectively dissolve P3OT, but not dissolve the block-copolymers. Since the F-P3OT block decreases the overall solubility of the polymers, this was easily achieved in the case of the polymer resulting from a 1:3 feed. Indeed, this diblock polymer being insoluble in chloroform and P3OT exhibiting excellent solubility in this solvent, the P3OT homopolymer was simply removed by Soxhlet extraction with chloroform.

**Figure 1:**
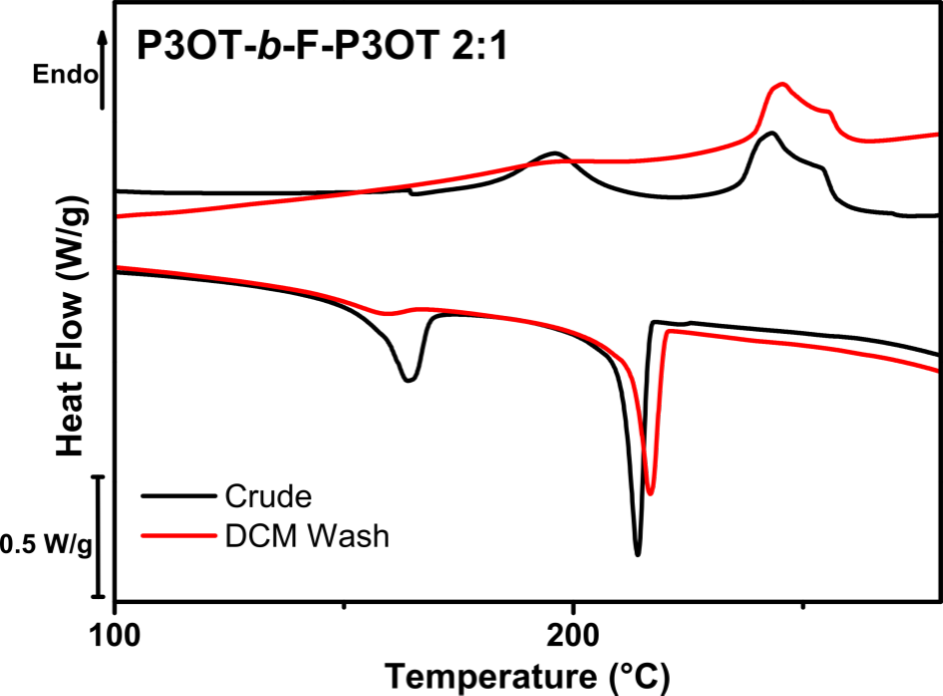
Differential scanning calorimetry thermogram (second cycle, 10 K/min) of P3OT-*b*-F-P3OT 2:1 before (black) and after (red) washing with dichloromethane. The reduction in the thermal transitions occurring below 200 °C, attributed to P3OT, indicate that a substantial amount of P3OT homopolymer was present in the crude polymer, but was removed by extraction.

On the other hand, the comparatively smaller difference in solubility between P3OT and the P3OT-*b*-F-P3OT resulting from a 3:1 feed meant that this approach was not feasible. In that case, washing with dichloromethane resulted in a demonstrable removal of P3OT homopolymer, as indicated by the differential scanning calorimetry (DSC) thermogram (see Figure 1). Indeed, the melting peak around 190 °C, apparent in the crude block copolymer and attributable to free P3OT, is clearly reduced upon washing. It is worth noting that shallow thermal transitions in the regions expected for P3OT are still present in this block copolymer, and this may be due to a small fraction of higher molecular weight P3OT that could not be fully removed by dichloromethane washing.

**Figure 2:**
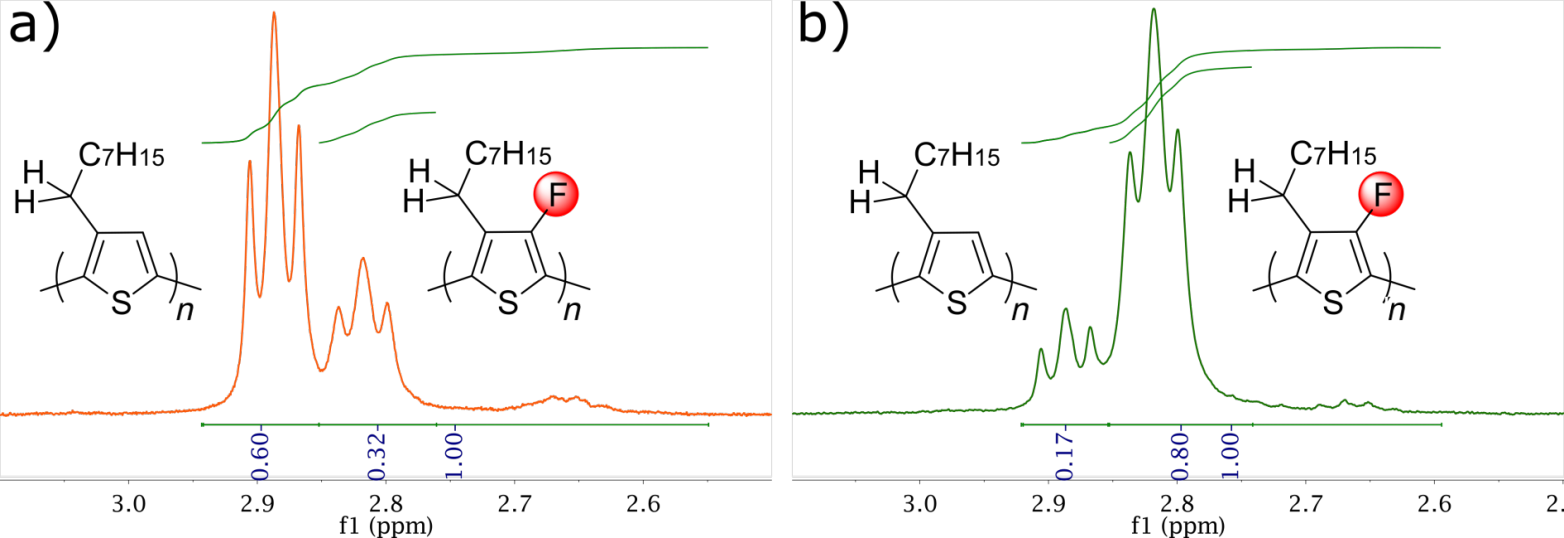
Selected region of the ^1^H NMR (*d*_2_-TCE at 403 K) of P3OT-*b*-F-P3OT produced with 3:1 (a) and 1:3 (b) monomer feed ratios. The signals correspond to the methylene protons adjacent to the thiophene ring.

The true relative block lengths were calculated from the ^1^H NMR spectra of the purified polymers, based on the relative intensities of the signals assigned to the methylene protons adjacent to the thiophene ring (Figure 2). Due to the reduced solubility of the polymers, the spectra were recorded in 1,1,2,2-tetrachloroethane-*d*_2_ at 403 K. The chemical shifts of the methylene protons for the fluorinated and non-fluorinated polymers are distinct, with the fluorinated block apparent as a triplet at 2.82 ppm, in very close agreement to the signal observed for the pure F-P3OT. In comparison, the non-fluorinated block occurs as a distinct triplet at 2.89 ppm, demonstrating the shielding effect of the *ortho*-fluorine. The ^19^F NMR (see Supporting Information File 1, Figures S1 and S2) shows a single peak in both cases, demonstrating that these polymers are indeed regioregular block copolymers with little mixed region within a polymer chain. Integration of the methylene regions in the ^1^H NMR indicates that the ratio of the two blocks deviates from the feed ratio, with a ratio P3OT-*b*-F-P3OT 2:1 and P3OT-*b*-F-P3OT 1:4 found (cf*.*, 3:1 and 1:3 feed ratios, respectively).

We note that the peaks around 2.65 ppm are likely related to regiochemical defects such as head-to-head or tail-to-tail couplings in the backbone [43], as well as the methylene region from the chain end [44]. Integration of this region with respect to the overall methylene region gives approximate regioregularites of 92 and 97% for P3OT-*b*-F-P3OT 2:1 and P3OT-*b*-F-P3OT 1:4, respectively. The higher regioregularity for the dominant F-P3OT block polymer may result from the fact that a single isomer is formed during the Grignard metathesis reaction for 2,5-dibromo-4-fluoro-3-octylthiophene [42], whereas the equivalent non-fluorinated monomer gives an approximate 4:1 mixture of regioisomers [45–46]. Although this mixture of isomers can afford good regularity under certain conditions, it can be expected to have a detrimental effect on regioregularity [47]. For this reason most block co-polymers utilize a regiochemically pure organomagnesium reagent prepared from 2-bromo-5-iodo-3-alkylthiophene [28].

The reason for the large discrepancy between the feed ratio and the true block lengths in P3OT-*b*-F-P3OT 2:1 may be attributed to unexpected chain termination during the growth of the P3OT block. This theory is consistent with the large amount of P3OT homopolymer that was removed from the crude product upon Soxhlet extraction, as indicated by DSC. In the case of P3OT-*b*-F-P3OT 1:4, the discrepancy likely arises from the fact that the activated M–H monomer is intrinsically comprised of ca. 20% of a regioisomer which is relatively unreactive towards KCTP when using 1,2-bis(diphenylphosphino)propane as ligand [32,46,48–49]. This results in a reduced effective concentration of **2** relative to **4**, since in the case of **4** the regioselectivity of the monomer activation is over 95% [42].

Gel-permeation chromatography (GPC) measurements in hot (80 °C) chlorobenzene gave the number average molecular weight (*M*_n_) of P3OT-*b*-F-P3OT 2:1 as 55 kg/mol against polystyrene standards. Although this is higher than the theoretical *M*_n_ (ca. 39 kg/mol for a monomer:catalyst ratio of 200:1), it is likely due to the removal of lower molecular weight oligomers during Soxhlet extraction as well as the known overestimation of molecular weight for polythiophenes when measured by GPC against polystyrene standards [50–51]. Accounting for the purification, the *M*_n_ of P3OT-*b*-F-P3OT 2:1 is in reasonable agreement with the ratio of H and F blocks as determined by ^1^H NMR, and the molecular weight of a crude sample taken prior to the addition of **4**, which showed an *M*_n_ of 31 kg/mol (theoretical *M*_n_ 29 kg/mol, Supporting Information File 1, Figure S3).

Despite having slightly better solubility than the homopolymer of F-P3OT, the solubility of P3OT-*b*-F-P3OT 1:4 in chlorobenzene was nevertheless too low to allow the molecular weight to be measured on our GPC instrument. A sample taken prior to the addition of **4** gave the molecular weight of the P3OT block as *M*_n_ 15 kg/mol (theoretical *M*_n_ 10 kg/mol), which would afford a final *M*_n_ at ca. 75 kg/mol when accounting for the ^1^H NMR ratios of H to F blocks.

### Optoelectronic properties

Our previous study on fluorinated poly(3-alkylthiophene) demonstrated that backbone fluorination leads to a ca*.* 0.3 eV increase in the ionisation potential (IP) compared to the non-fluorinated polymer [42]. In order to probe this effect in block copolymers, the IPs of P3OT-*b*-F-P3OT 2:1 and P3OT-*b*-F-P3OT 1:4 were measured by photoelectron spectroscopy in air (PESA). The results suggest that the IP in this system is mostly defined by the most abundant block. Indeed, the 2:1 block copolymer has an IP of 4.83 eV, slightly higher than that of P3OT (4.70 eV) measured by the same technique [42], while the 1:4 copolymer had an IP of 5.03 eV, which is within experimental error (±0.05 eV) of the IP of F-P3OT (4.99 eV) [42].

To investigate the influence of the block compositions on the optical properties, the UV–visible absorption spectra of thin films spin-cast from hot 1,2,4-trichlorobenzene were measured. The thin film UV–visible absorption of polythiophene derivatives can provide some information about the molecular order of the polymer chains, via the interpretation of the vibronic shoulders [52–55]. In the case of mixtures and block copolymers this is somewhat complicated by the different overlapping absorption profiles of the components. The absorption spectrum of a polymer blend typically corresponds to the sum of the absorptions of the component polymers (assuming complete phase separation occurs). Making use of complementary absorption profiles for optimized harvesting of the solar spectrum is thus one of the main advantages of all-polymer photovoltaic devices [19,56–58]. The absorption spectra of block copolymers vary according to the system studied and processing conditions, since spontaneous phase separation is more difficult than in the case of polymer blends due to the chromophores being tethered. For example, in a polythiophene-polyselenophene block copolymer, isothermal recrystallization of the film results in an absorption profile that perfectly matches the linear combination of the homopolymers [26]. On the other hand, the absorption spectra of as spun block copolymers of P3HT with an analogue containing a ketone-functionalized side chain do not seem to linearly correlate to the composition ratio [59–60].

**Figure 3:**
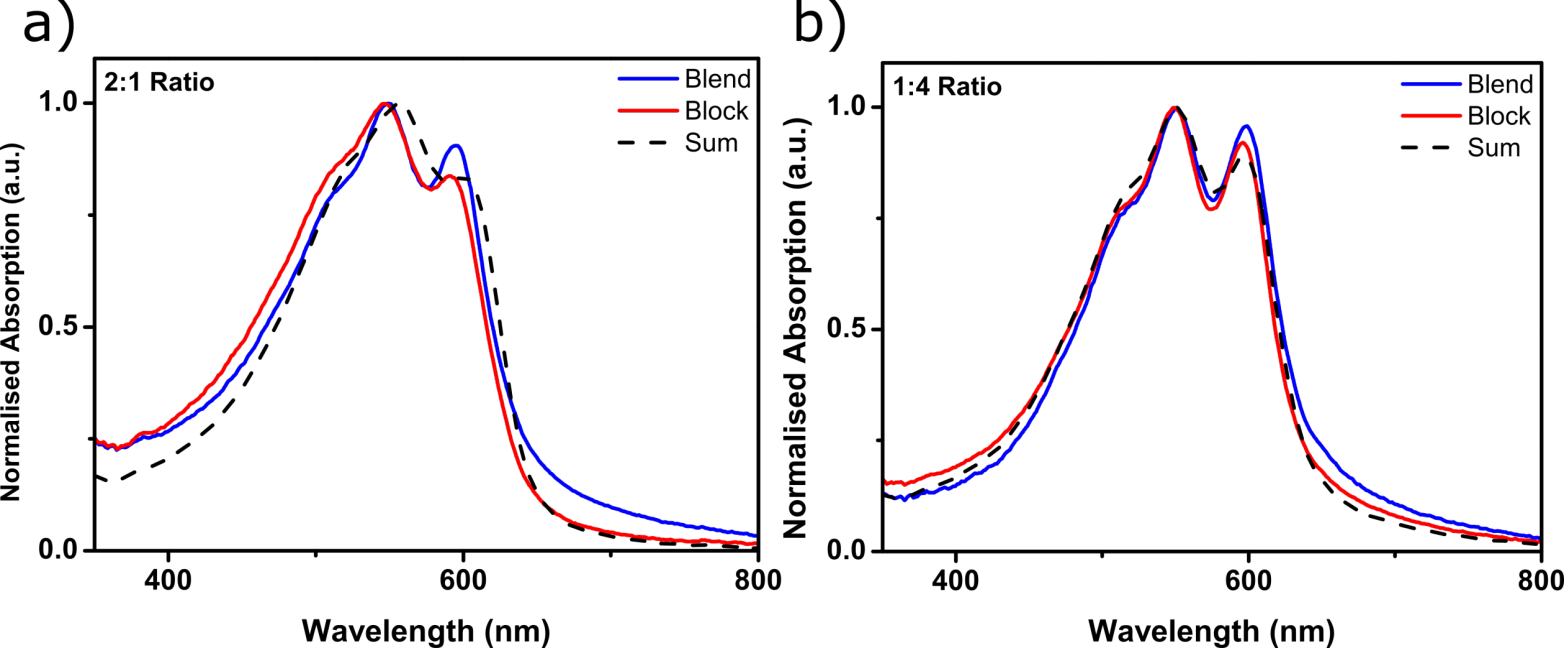
Thin film UV–visible absorption spectra of 2:1 (a) and 1:4 (b) P3OT-*b*-F-P3OT polymers (red line), overlaid with the UV–visible absorption spectra of a blend of P3OT and F-P3OT in the same ratio (blue line). The dotted line indicates the corresponding sum of the UV–visible absorption of P3OT and F-P3OT, weighted according to the composition. Thin films were spin cast from hot 1,2,4-trichlorobenzene.

**Figure 4:**
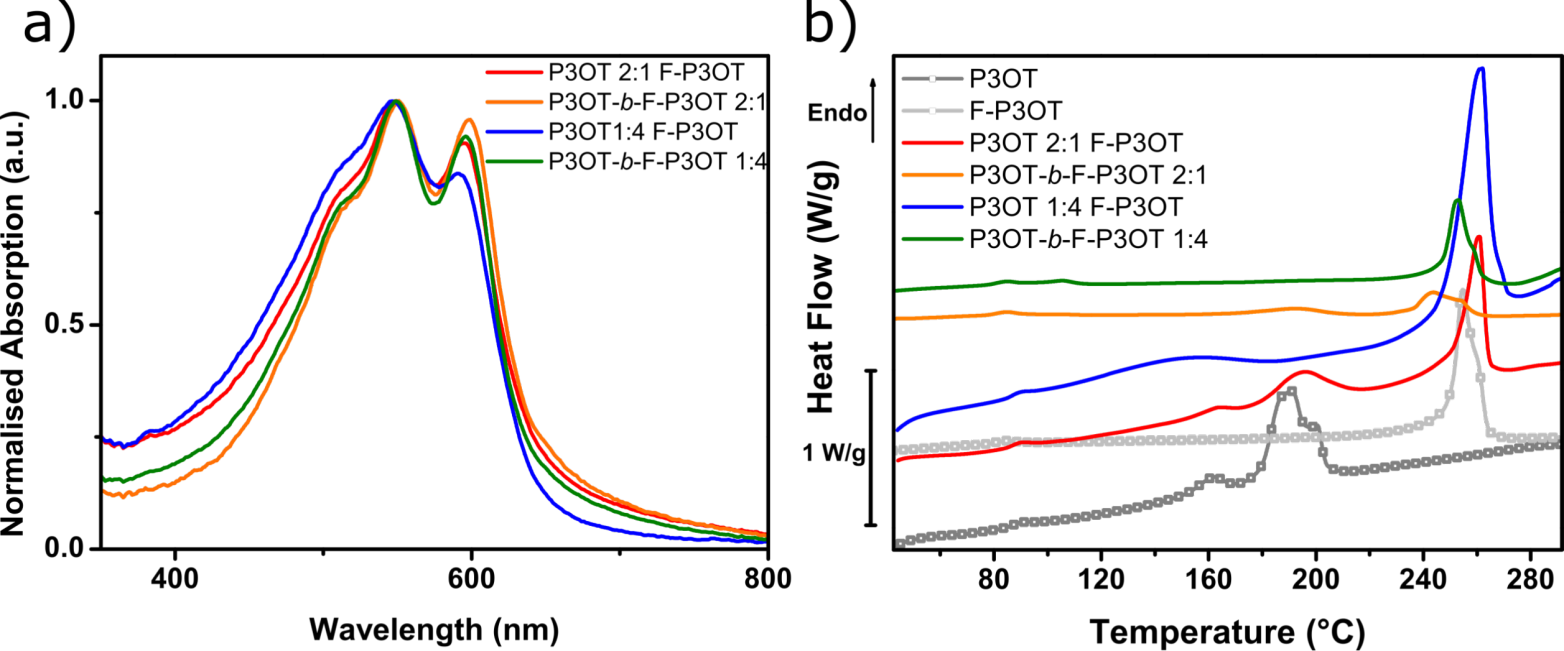
Overlay of thin film UV–visible absorption spectra (a) of the two block copolymers and the corresponding P3OT/F-P3OT blends. Thin films were spin cast from hot 1,2,4-trichlorobenzene. Differential scanning calorimetry thermograms (b) of dropcast films of the same samples. For comparison, thermograms of P3OT and F-P3OT as dropcast films are also included. Only the heating curve (first cycle, 10 °C/min) is shown. Note that the peak observed at ca. 85 °C in all traces is an artefact resulting from the instrument.

Figure 3 shows the absorption spectra of P3OT-*b*-F-P3OT in 2:1 and 1:4 ratios, as spun (see Figure 4 for overlay of both block copolymers and their corresponding blends). Also presented in Figure 3 are the absorption profiles of spin coated blends of P3OT and F-P3OT in the same ratios, as well as the weighted linear combination of the as spun absorption spectra of pure P3OT and F-P3OT polymers. All films were spin cast from hot 1,2,4-trichlorobenzene for comparison purposes. In the 2:1 ratio, all absorption profiles appear subtly different, both in terms of the peak positions and the vibronic structure. The block and blend films exhibit a slightly blue-shifted absorption maxima compared to the linear combination of homopolymers, as well as a more pronounced vibronic structure. Since F-P3OT homopolymer has a blue-shifted absorption with more vibronic structure than P3OT (vide infra), it can be deduced that the absorption profile of the block and blends are dominated by F-P3OT sections of the mixture, despite it having a lower concentration. Unsurprisingly therefore, the films in 1:4 ratios have approximately the same absorption profile, with the peak positions being nearly identical to the F-P3OT homopolymer. Although the multicomponent nature of the systems likely complicates the interpretation of the vibronic structure in relation to the order in the thin film, it is worth noting that the vibronic structure of the block copolymers in both cases is less pronounced than for the corresponding blend. Considering the aformentioned link between the vibronic structure and aggregation, this likely indicates less inter- and intrachain coupling, and possibly a frustration of the crystallization and phase separation in the case of these block copolymers.

### Thermal behavior

#### Differential scanning calorimetry

Fluorination of P3OT has previously been shown to result in a 50–60 °C increase in the melting and crystallization temperatures, an effect that was predominantly attributed to the increased backbone planarity and rigidity in combination with the increased aggregation it engenders [42]. In order to probe the crystallization behavior of the block copolymers and blends in thin films, DSC was therefore performed. Rather than investigating the melting transitions of bulk polymer powders, which are not always representative of films cast from solution, films were cast and then scraped off the substrate to be measured. Due to the poor solubility of the polymers with high fluorine content, the film thicknesses achievable when spin coating were low, and dropcasting onto a hot substrate was therefore used. The DSC thermograms of the resulting films are shown in Figure 4b.

Immediately obvious from these thermograms is the prominence of a high-temperature (>240 °C) melting transition, in all cases except pure P3OT (ca. 180 °C). This transition can reasonably be attributed to the fluorinated block/polymer portion, due to its close proximity with the melting point of the F-P3OT homopolymer. As in the UV–visible absorption spectra, the domination of F-P3OT features is evident, particularly in the 2:1 blend which, despite containing twice the amount of P3OT than F-P3OT, still shows a much sharper and evident higher temperature melting transition characteristic of F-P3OT. The fact that the 2:1 blend undergoes two melts closely corresponding to each component polymer suggests that it has sufficient phase separation to allow both polymers to crystallize in discrete domains. On the other hand, in the 1:4 blend the melting transition of P3OT is absent. These observations may be explained by the greater melting enthalpy of the crystalline phase of F-P3OT compared to P3OT (28 and 17 J/g, respectively [42]), which could result in a masking of the P3OT melt in the baseline. However, the apparent suppression of the P3OT melt could also be explained by some degree of frustration of the P3OT crystallization, likely due to the earlier precipitation of the less soluble F-P3OT during the film formation.

P3OT-*b*-F-P3OT in a 1:4 ratio displays much the same melting behavior as the corresponding blend, although the onset of melting is slightly lower. This may be explained by the short P3OT segments, which although they may be too short to cause phase separation and crystallize themselves, likely cause some disruption to the crystallization of the F-P3OT segment [59,61]. In the case of P3OT-*b*-F-P3OT 2:1, there are two distinct melting transitions which match those of P3OT and F-P3OT. Since it cannot be said with full confidence that all P3OT homopolymer was removed during purification, it is unclear whether these two transitions indicate phase separation and therefore separate melting transitions for each block, or are simply due to the residual P3OT impurity. However, the low melting enthalpies of both peaks and the lower melt onset of the F-P3OT block suggests that crystallization is frustrated even for the fluorinated block.

#### Influence of annealing temperature on UV–visible absorption

**Figure 5:**
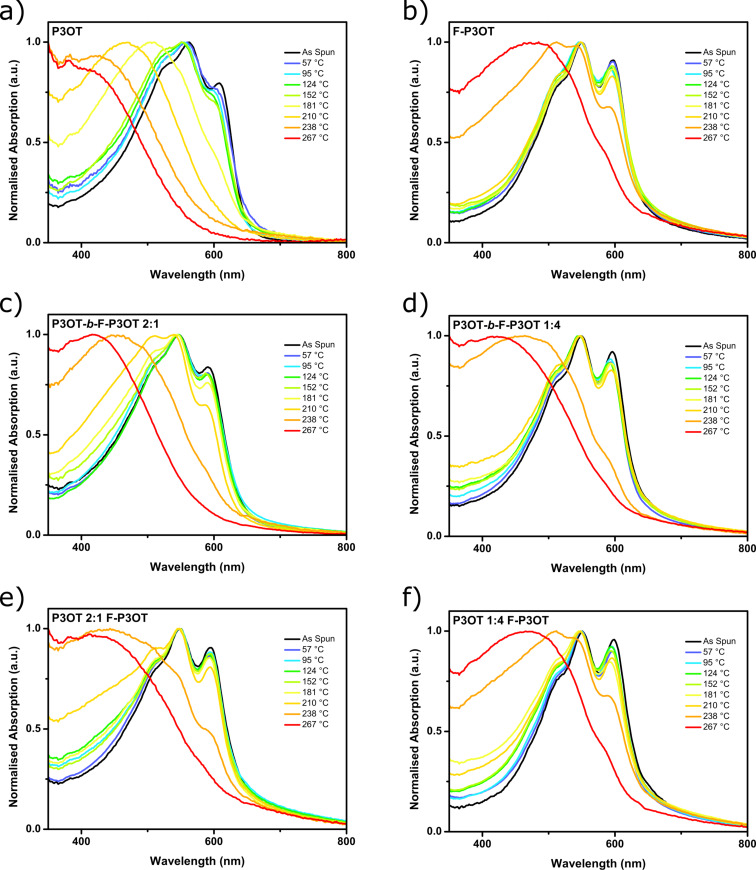
Thin-film UV–visible absorption spectra of a) P3OT, b) F-P3OT, c) P3OT-*b*-F-P3OT 2:1, d) P3OT-*b*-F-P3OT 4:1, e) P3OT/F-P3OT blend in a 2:1 ratio, and f) P3OT/F-P3OT blend in a 4:1 ratio. Films were annealed at the temperatures indicated for 20 min, then quench cooled and the UV–visible absorption spectrum measured. The same films were used for subsequent annealing at higher temperatures. Thin films were spin cast from hot 1,2,4-trichlorobenzene.

The influence of annealing temperature on the optical absorption spectra of the polymers was also investigated. Here films were annealed at the specified temperature for 20 minutes on a hotplate under argon before rapid quench cooling. The evolution of the thin film UV–visible absorption spectra with increasing annealing temperature (see Figure 5) partly reflects the thermal behavior of the samples as observed by DSC. In all cases, the low energy shoulder decreases in intensity with increasing annealing temperature, until a critical point when the absorption profile dramatically blue shifts and loses all or most of its vibronic structures. The broad absorption exhibited by the samples beyond this critical point is reminiscent of the absorption of fully solvated polythiophene derivatives, and therefore suggests that beyond this point the polymers have essentially been quenched in the melt state with disordered backbones and little inter- and intrachain coupling. This is further supported by the fact that these critical temperatures are in good agreement with the melt temperatures exhibited by the dropcast samples (see Table 1).

**Table 1:** Melting point of dropcast films taken as the maximum (onset in parentheses), and critical annealing temperature of spin cast films at which vibronic structure is mostly lost from the UV–visible absorption.

			P3OT-*b*-F-P3OT		P3OT blend with F-P3OT	
	
	P3OT	F-P3OT	2:1	1:4	2:1	1:4

Melting point (°C)	190 (177)	254 (242)	193 (178) / 243 (235)	253 (243)	194 (180) / 261 (240)	261 (241)
Critical annealing temperature (°C)	181	267	238	238	238	267

The critical temperature in the case of P3OT is near 181 °C, which is above the onset of melting transition, and all the vibronic structure is lost from the absorption when annealed well above the melt at 210 °C. Higher temperature annealing simply shifts the absorption to higher energy, consistent with more disordered backbones as is observed in heated solutions of polythiophenes [42]. In the case of F-P3OT, annealing just under the melting onset (238 °C) leads to a significant shift in the vibronic structure. The large increase in the intensity of the high-energy part of the spectrum suggests an increase in the proportion of disordered polymer [55,62], as expected at the onset of a melt, while the decreased intensity of the 0–0 shoulder may be indicative of a greater degree of interchain coupling and therefore the formation of H-aggregates [63]. Annealing at temperatures higher than the melt disrupts these aggregates, and although minor vibronic shoulders are still present, these are likely due to some degree of aggregation during the cooling prior to the measurement of the spectrum.

The blend and block copolymers behave in a similar manner to each other, with dramatic shifts in the vibronic structure near the onset of the melting transition and then a blue shift and loss in the vibronic structure when annealed beyond the highest temperature melt. In the 2:1 ratio samples, phase separation is suggested by the additional small changes in the vibronic structure seen when annealing is performed at 210 °C, beyond the low temperature transitions associated with P3OT domains melting, but below the onset of the F-P3OT melt. In the 1:4 ratio samples, the dominance of F-P3OT in the absorption profile means such small changes are not obvious. The only suggestion of P3OT chains melting is the small increase in absorption that is seen in the high-energy (ca*.* 400 nm) region of the spectra. The fact that this progression is more pronounced than in the case of pure F-P3OT may indicate phase separation since pure P3OT domains would be fully melted and disordered at such temperatures, and thus result in increased absorption in the high-energy region.

It is interesting to note that the vibronic structure is more resistant to higher annealing temperatures in the blends, perhaps indicating that in the block copolymers the disorder of the melted P3OT sections help break up the F-P3OT rich aggregates, or that these polymers do not phase separate as much, and may even produce co-crystals with lower melt onsets.

#### Temperature dependent Raman spectroscopy

Having probed the effect of temperature on the intermolecular interactions of the samples using DSC and UV–visible absorption spectroscopy, we performed in situ Raman spectroscopy measurements, similar to those previously carried out on P3OT and F-P3OT [42] in order to observe the evolution of intramolecular order with temperature, and compare the block copolymers with the polymer blends. The room temperature Raman spectra (see Supporting Information File 1, Figures S4–S6) show four strong peaks in the range 1300 cm^−1^ to 1700 cm^−1^, associated with stretching modes of the conjugated polymer backbone. These peaks can be readily assigned to the P3OT and F-P3OT blocks of the polymer chain by comparison with the homopolymer spectra. We identify the 1381 cm^−1^ and 1446 cm^−1^ peaks as the stretching modes of the P3OT block, corresponding with symmetric C–C and C=C collective bond stretches. In the F-P3OT block, a similar pair of modes is observed with peaks at 1416 cm^−1^ and 1491 cm^−1^, whose natures appear to be comparable to the non-fluorinated case and so can also be described as C–C and C=C collective stretches, respectively [42].

The Raman spectra of both the 2:1 diblock and blend films resemble a linear combination of the spectra for the neat homopolymers, where the peak positions are the same as for the homopolymers, but the intensities of the F-P3OT modes are stronger than those of the P3OT modes, contrary to what is expected from the molar ratio. This indicates that the Raman scattering cross-sections for these modes in the F-P3OT polymer are greater than for the corresponding modes in P3OT (by a factor of ≈2). As a result, the Raman spectra for both the 1:4 diblock and blend samples are dominated by the F-P3OT peaks, with minimal distinct contributions from the P3OT modes.

The temperature dependent Raman spectra of the pure homopolymers have been reported previously, and the blend and diblock samples display similar trends [42]. Specifically, as the temperature increases, all four main peaks move towards lower Raman shifts; the overall scattering Raman scattering intensity gradually reduces; and the intensities of the C–C modes (1381 cm^−1^ and 1416 cm^−1^) reduce with respect to the corresponding C=C modes (1446 cm^−1^ and 1491 cm^−1^, respectively). A distinct transition is observed in the Raman spectra at around 260–270 °C for P3OT and at 300–310 °C for F-P3OT, where the Raman scattering intensity reduces dramatically and the C=C peaks move towards higher Raman shifts (1469 cm^−1^ for P3OT and 1505 cm^−1^ for F-P3OT). These effects are found to be largely reversible upon cooling (see Supporting Information File 1, Figure S7).

Below the transition, the observed reduction in Raman scattering intensity is consistent with a thermally-induced reduction in the ground vibrational state population, and the shifts in Raman peak position are associated with a combination of anharmonic ‘softening’ of the vibrational modes as well as conformational planarization of the polymer backbone [64–65]. The observed transition at 260–310 °C was previously assigned to overcoming the energetic barrier to rotation around the inter-ring C–C bond, resulting in a loss of effective π-conjugation as well as the Raman peaks associated with highly ordered polymer phases (in particular the lower energy contribution to the main C=C peak) [42].

**Figure 6:**
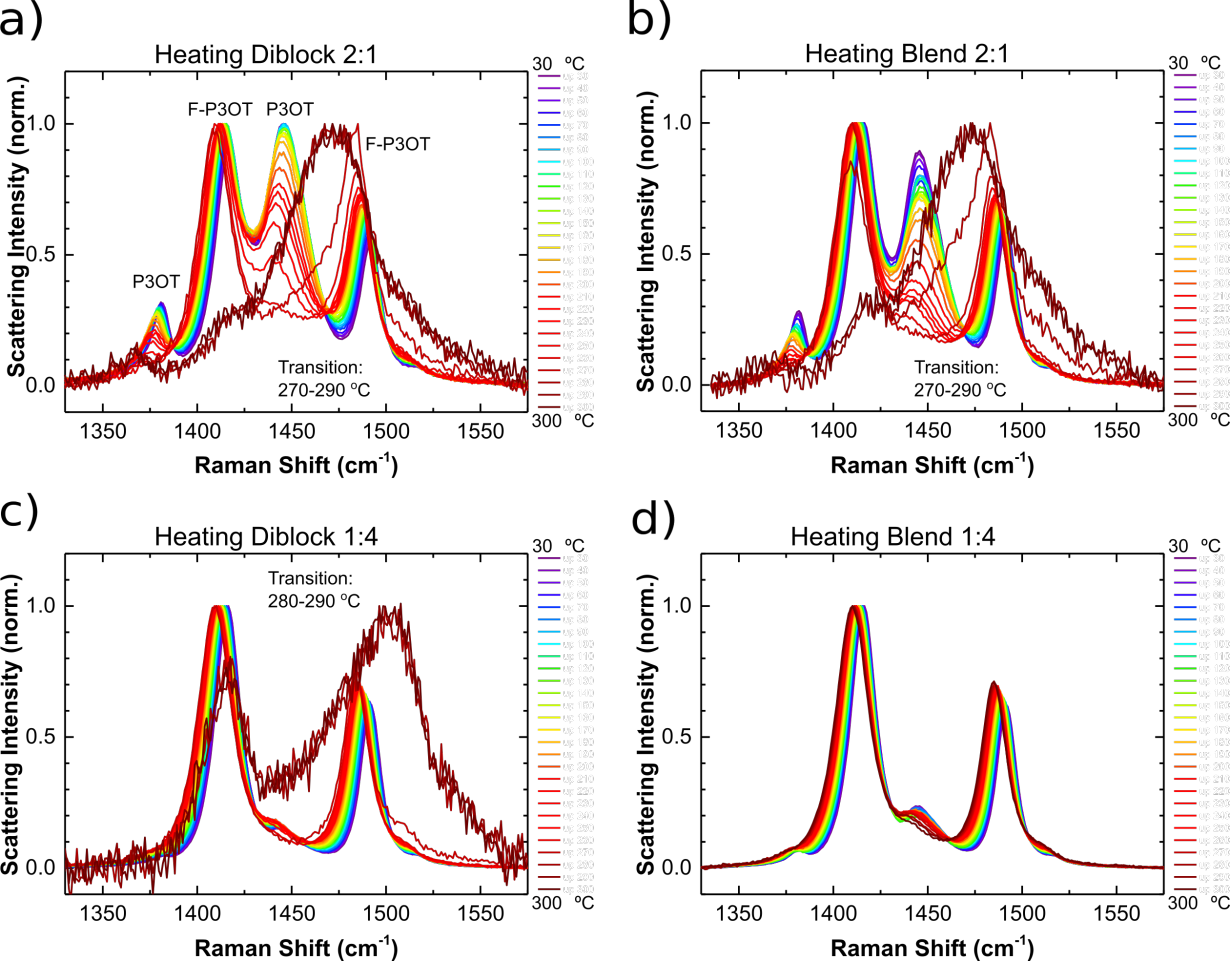
Temperature dependent Raman spectra measured during heating showing the main C–C and C=C stretches of a) P3OT-*b*-F-P3OT 2:1, b) P3OT/F-P3OT blend in a 2:1 ratio, c) P3OT-*b*-F-P3OT 4:1, and d) P3OT/F-P3OT blend in a 4:1 ratio. The main peaks are assigned to the fluorinated (F-P3OT) and nonfluorinated (P3OT) segments in a) based on the homopolymer spectra [42].

The temperature dependent Raman spectra of the diblock and blend films presented in Figure 6, show broadly the same features and trends as their component homopolymers: all four main peaks move towards lower Raman shifts with increasing temperature and the intensity of the C–C peaks also decrease with respect to the C=C peaks. The same transition is also observed in the range of 270–290 °C, for all of the samples, except for the 4:1 blend film. Since the Raman scattering from all of these samples is dominated by the F-P3OT component, the P3OT thermal transition temperature range is not distinct, however, it is striking that the F-P3OT transition is observed at a lower temperature for both diblocks and the 1:2 blend samples than for neat F-P3OT. In fact, the observed temperature range of the transition is comparable with that of the neat P3OT homopolymer (≈260 °C), which suggests that the event occurring within the P3OT chains promotes a similar event in the F-P3OT sections. In the block copolymers, where the F-P3OT and P3OT segments are tethered and electronically conjugated this is perhaps less surprising. However the 2:1 blend, which shows evidence of phase separation from the DSC measurements, also exhibits a 20–40 °C depression in the temperature of the Raman transition of the F-P3OT signals. The conclusion from this observation is that the structural event that leads to the Raman transition may be somewhat cooperative, even between polymer domains.

**Figure 7:**
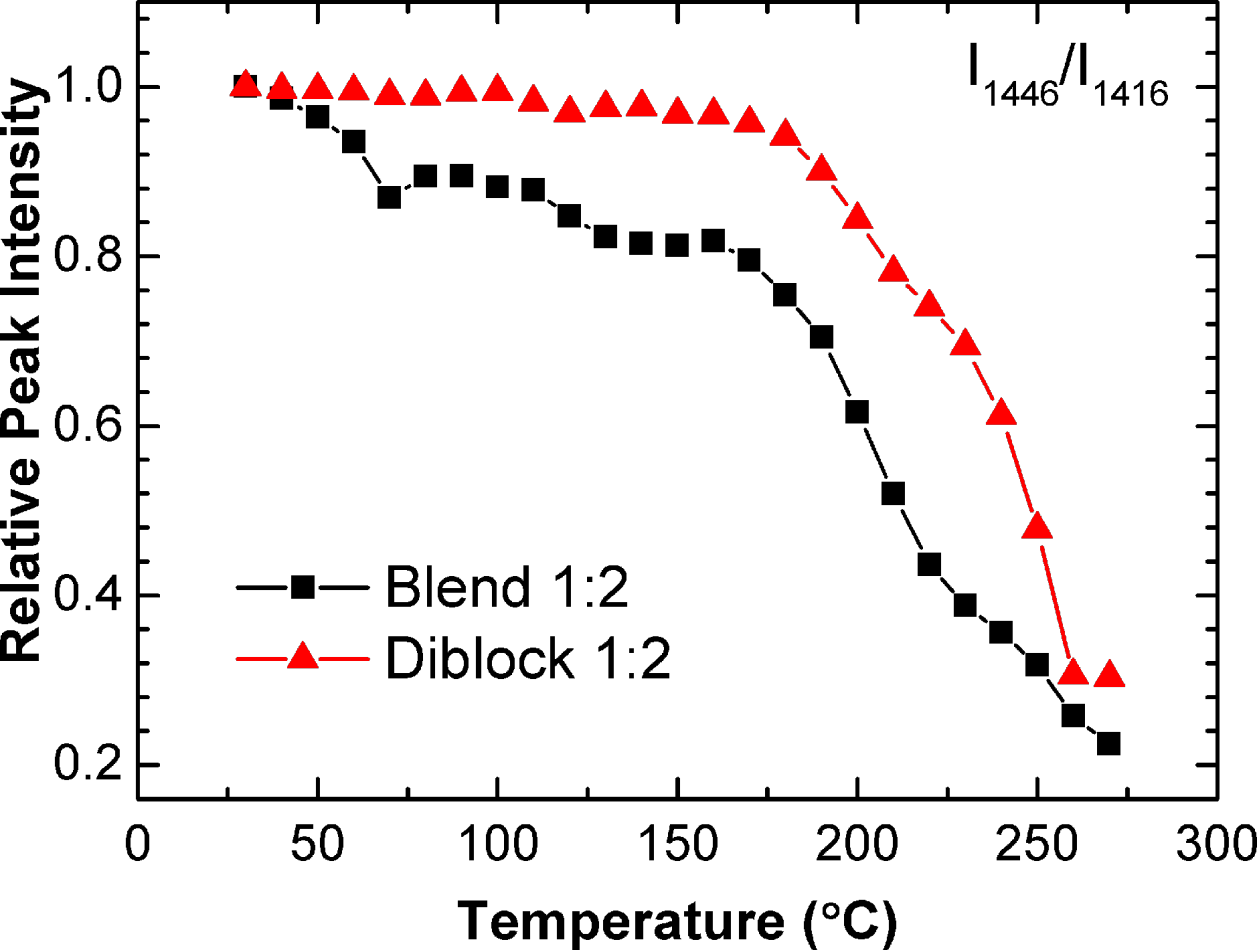
Influence of temperature on the relative Raman scattering intensities of the P3OT (1446 cm**^−^**^1^) and F-P3OT (1416 cm**^−^**^1^) C=C bond stretching modes. The block copolymer appears to have a constant relative intensity until *ca.* 175 ºC, while the blend appears less stable, showing decay almost immediately upon heating.

With previous reports of block copolymers stabilizing morphology (see Introduction), it is interesting to note that the P3OT-*b*-F-P3OT 2:1 exhibits greater thermal stability than the corresponding blend, when probed using the temperature dependent Raman spectrum. In this case, we consider the intensities of the strongest peaks measured for the P3OT (1446 cm^−1^) and F-P3OT chains (1416 cm^−1^). Since the intensities of these peaks correspond with the degree of conjugated backbone planarity for their respective chain segments, the ratio I_1446_/I_1416_ gives an indication of the relative planarity of the P3OT part compared with the F-P3OT. This ratio is plotted as a function of temperature for both the 2:1 blend and diblock films in Figure 7. We find that this ratio drops consistently with temperature for the 2:1 blend film up to ca. 175 °C, where a more dramatic decrease is observed. In contrast, for the diblock copolymer, the ratio remains almost constant up to a similar temperature (ca. 175 °C) before decreasingly more sharply. At temperatures above ≈250 °C the intensity ratios for both films are similar. It is noteworthy, that the onset of the change in decay rate of this ratio at around 175 °C occurs at a similar temperature to the onset of the P3OT melting transition in the DSC thermogram. The increased thermal stability of the diblock over the blend sample in this case does not appear to affect the melting temperature significantly, rather the stabilization relates to the molecular conformational order and particularly the planarity of the conjugated polymer backbone. This result indicates that the diblock copolymer is able to maintain ordered P3OT domains to a higher temperature than the equivalent blend film, and suggests that this is a viable strategy for enhancing morphological stability at elevated temperatures.

## Conclusion

In order to investigate the effects of partial planarization of the polymer backbone through fluorination on the physical and optoelectronic properties of the P3OT system, two block copolymers were synthesized via sequential monomer addition under by Kumada catalyst transfer polymerisation (KCTP) conditions. Diblock polymers with a large and small F-P3OT block were therefore synthesized. The P3OT-*b*-F-P3OT polymers were compared to their analogous blends of the corresponding homopolymers.

The F-P3OT block dominates the UV–visible absorption spectra, thermal behavior and Raman spectra, both in block copolymers and blends. The crystallization in the block copolymers appears to be slightly impeded, as suggested by the depressed melting point and reduced vibronic structure in the UV–visible absorption spectrum. The highest melting transition temperatures for each polymer or blend matches well with the critical annealing temperature at which the thin-film UV–visible absorption spectra exhibit a dramatic blue-shift in the absorption, and a loss in vibronic structure. The block copolymers have marginally lower critical temperatures than their blend analogues, perhaps further signifying impeded crystallization due to the P3OT segments disrupting the F-P3OT domains.

This is further corroborated by the fact that a thermal event, evident in the Raman spectra, attributed to a significant change in intramolecular order occurs in 3 of the 4 mixed systems at the same temperature as for pure P3OT (ca*.* 260–270 °C), even for F-P3OT segments (310 °C for pure polymer). Only in the 1:4 P3OT/F-P3OT blended film are the temperature dependent Raman spectra unaffected by the presence of P3OT, as is the case in the behavior measured using UV–visible absorption spectroscopy and DSC.

In the case of the 2:1 block copolymer, the temperature dependence of the Raman signals attributed to P3OT-rich polymer chains is found to be substantially different to the corresponding blend at temperatures below the thermal transition. While the intensity of the thiophene C=C stretching mode steadily decreases with increasing temperature in the case of the blend, it remains constant in the block copolymer until temperatures approaching the melting point of P3OT.

All the experimental data collected suggests that the increased propensity of F-P3OT to aggregate translates to a domination of the optoelectronic and temperature dependent properties of the thin film, even as a minority component. P3OT and F-P3OT blends are also found to behave like a mostly phase-separated system, while the block copolymers exhibit signs of frustrated crystallization and self-assembly. It is likely that optimizations in the processing techniques, thermal annealing and solution self-assembly may help to improve this self-assembly.

## Experimental

### General

Reagents and chemicals were purchased from commercial sources such as Aldrich and Acros etc. unless otherwise noted. P3OT and F-P3OT and the corresponding monomers were synthesized according to our previous work [42]. The batch of P3OT used in this study had *M*_n_ 26 kg/mol, *M*_w_ 33 kg/mol (as measured by GPC in chlorobenzene at 80 ºC), while the F-P3OT used *M*_n_ 53 kg/mol, *M*_w_ 98 kg/mol (as measured by HT-GPC in 1,2,4-trichlorobenzene at 130 °C).

All reactions were carried out under argon using solvents and reagents as commercially supplied, unless otherwise stated. ^1^H and ^19^F NMR spectra were recorded on a Bruker AV-400 (400 MHz), using the residual solvent resonance of CDCl_3_ or *d*_2_-1,1,2,2-tetrachloroethane and are given in ppm. Number-average (*M*_n_) and weight-average (*M*_w_) were determined by Agilent Technologies 1200 series GPC running in chlorobenzene at 80 °C, using two PL mixed B columns in series, and calibrated against narrow polydispersity polystyrene standards.

Films for PESA, UV–visible absorption and Raman spectroscopy, were prepared by spin-coating from hot (ca. 150 °C) solution in 1,2,4-trichlorobenzene (5 mg/mL) at 3000 rpm for 2 minutes. Dropcast films for DSC measurements were prepared by dropcasting a hot (ca. 150 °C) 5 mg/mL solution in 1,2,4-trichlorobenzene onto hot glass substrates (ca*.* 120 °C) and letting the solvent evaporate. The film was then scraped off using a knife and the powder used for DSC analysis.

UV–visible absorption spectra were recorded on a UV-1601 Shimadzu UV–vis spectrometer. Each film was annealed (under flow of Ar) for 20 min at the lowest temperature, then cooled on a surface at room temperature before measuring the UV–visible absorption spectrum. The same film was then annealed and spectrum measured in a similar way for each subsequent temperature.

Photo electron spectroscopy in air (PESA) measurements were recorded with a Riken Keiki AC-2 PESA spectrometer with a power setting of 5 nW and a power number of 0.5. Samples for PESA were prepared on glass substrates by spin-coating.

Differential scanning calorimetry (DSC) measurements: ≈2 mg material was used for the DSC experiments, which was conducted under nitrogen at a scan rate of 10 °C/min with a TA DSC-Q20 instrument.

Raman spectra were measured using a Renishaw inVia Raman spectrometer with 785 nm diode laser excitation. Laser power at the sample was 130 mW focussed to a 40 μm^2^ area. The photoluminescence background was subtracted from the spectra using a polynomial baseline and then the spectra were normalized to the main peak. A Linkam THMS600 hot-cold cell purged with nitrogen was used to prevent polymer degradation as well as to control the temperature of the sample. For room temperature measurements the total laser exposure time was 25 s, the exposure time for temperature dependent spectra was 10 s. Starting from room temperature, the sample was heated at 10 °C/min to 300 °C, then cooled at the same rate. The temperature was held for 1 minute at every 10 °C interval in order to measure spectra.

### Typical procedure for the synthesis of Grignard monomer

To a solution of 2,5-dibromo-3-octylthiophene (361.2 mg, 1.02 mmol) in dry THF (2.86 mL) at room temperature was added isopropylmagnesium chloride lithium chloride complex (0.78 mL, 1.3 M in THF) dropwise. After 30 min, the resulting Grignard monomer solution (0.28 M in THF) was ready for use as indicated by the near total consumption of starting material (<3% remaining by quenching a sample with methanol and analysing by GC–MS).

### Synthesis of P3OT-*b*-F-P3OT 2:1

In a sealed dry 2–5 mL microwave vial charged with dichloro(1,3-bis(diphenylphosphino)propane)nickel (2.27 mg, 0.5 mol %) was added Grignard solution freshly prepared from 2,5-dibromo-3-octylthiophene (2.25 mL, 0.28 M in THF), and the reaction mixture was stirred at 40 °C for 1 h. GPC analysis of an aliquot quenched with methanol/HCl indicated *M*_n_ 31 kg/mol, *M*_w_ 43 kg/mol. A Grignard solution freshly prepared from 2,5-dibromo-3-fluoro-4-octylthiophene (0.75 mL, 0.28 M) was added to the reaction mixture, and the reaction heated to 70 °C for 2 h before being poured into methanol (200 mL) acidified with a few drops of conc. HCl. The precipitate was filtered through a cellulose thimble, and the solid purified by Soxhlet extraction with methanol, acetone, and hexane. In order to determine if substantial amounts of P3OT homopolymer still remaining in the sample, a DSC was run on a sample, and after confirmation that this was indeed the case, the solid was further washed with dichloromethane and finally extracted with chloroform, before precipitation into methanol and filtration. The resulting solid was dried under vacuum to give P3OT-*b*-F-P3OT 2:1 (50 mg, 25%). *M*_n_ 55 kg/mol, *M*_w_ 60 kg/mol; ^1^H NMR (400 MHz, TCE-*d_2_*, 403 K, δ) 7.05 (s, 1H), 2.94–2.85 (m, 1.8H), 2.85–2.77 (m, 0.9H), 1.90–1.66 (m, 3H), 1.56–1.35 (m, 15H), 1.03–0.90 (m, 4.2H); ^19^F NMR (376 MHz, TCE-*d_2_*, 403 K, δ) −122.94 (s).

### Synthesis of P3OT-*b*-F-P3OT 1:4

In a sealed dry 2–5 mL microwave vial charged with dichloro(1,3-bis(diphenylphosphino)propane)nickel (2.27 mg, 0.5 mol %) was added Grignard solution freshly prepared from 2,5-dibromo-3-octylthiophene (0.75 mL, 0.28M in THF), and the reaction mixture was stirred at 40 °C for 1 h. GPC analysis of an aliquot quenched with methanol/HCl indicated *M*_n_: 15 kg/mol, *M*_w_: 18 kg/mol. A Grignard solution freshly prepared from 2,5-dibromo-3-fluoro-4-octylthiophene (2.25 mL, 0.28 M) was added, and the reaction heated to 70 °C for 2 h before being poured into methanol (200 mL) acidified with a few drops of conc. HCl. The precipitate was filtered through a cellulose thimble, and the solid purified by Soxhlet extraction with methanol, acetone, hexane and chloroform. The solid was dried and reprecipitated from 1,2,4-trichlorobenzene into methanol and filtered. The solid was dried under vacuum to give P3OT-*b*-F-P3OT 1:4 (104 mg, 64%). Molecular weight could not be measured due to lack of solubility. ^1^H NMR (400 MHz, TCE-*d_2_*, 403 K, δ) 7.05 (s, 1H), 2.93–2.86 (m, 1.8H), 2.85–2.76 (m, 8.3H), 1.91–1.64 (m, 10.9H), 1.58–1.27 (m, 56.9H), 1.06–0.89 (m, 15.1H); ^19^F NMR (376 MHz, TCE-*d_2_*, 403 K, δ) −122.94 (s).

## Supporting Information

^1^H and ^19^F NMR of P3OT-*b*-F-P3OT 2:1 and P3OT-b-F-P3OT 4:1, gel permeation chromatography trace for P3OT-*b*-F-P3OT 2:1, room temperature Raman spectra of P3OT and F-P3OT, room temperature Raman spectra of P3OT-*b*-F-P3OT 2:1 and P3OT-*b*-F-P3OT 1:4, room temperature Raman spectra of blends of P3OT and F-P3OT in 2:1 and 1:4 ratios, and temperature dependent Raman spectra measured during cooling.

File 1 Additional spectra.
