# Supplementary material for: Comparing blends and blocks: Synthesis of partially fluorinated diblock polythiophene copolymers to investigate the thermal stability of optical and morphological properties
Source: Beilstein J Org Chem. 2016 Oct 10;12:2150–63. doi: 10.3762/bjoc.12.205 (PMC5082479; doi:10.3762/bjoc.12.205)
Supplement: File 1 — Additional spectra. [file Beilstein_J_Org_Chem-12-2150-s001.pdf]

Supporting Information

for

Comparing blends and blocks: Synthesis of partially fluorinated diblock polythiophene copolymers to investigate the thermal stability of optical and morphological properties

Pierre Boufflet,[1] Sebastian Wood,[2] Jessica Wade,[2] Zhuping Fei,[1] Ji-Seon Kim,[2] and Martin Heeney*[1]

Address: [1]Department of Chemistry and Centre for Plastic Electronics, Imperial College London, Exhibition Rd, London, SW7 2AZ, U.K. and [2]Department of Physics and Centre for Plastic Electronics, Imperial College London, London, SW7 2AZ, U.K.

Email: Martin Heeney – m.heeney@imperial.ac.uk

* Corresponding author

Additional spectra

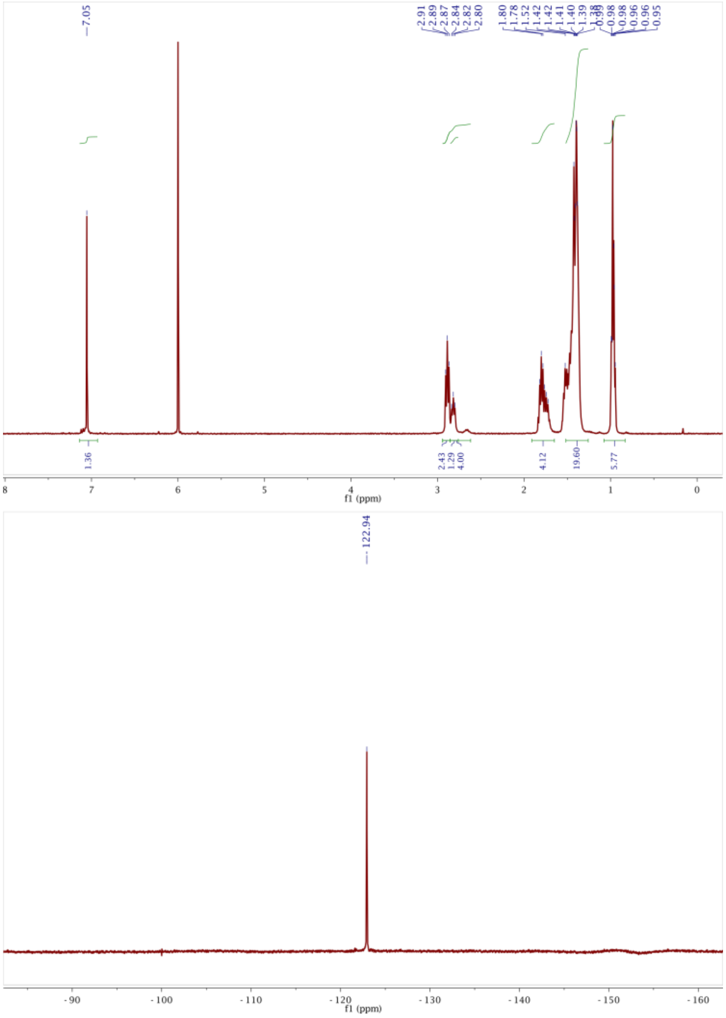

Figure S1: NMR P3OT-*b*-F-P3OT 2:1.

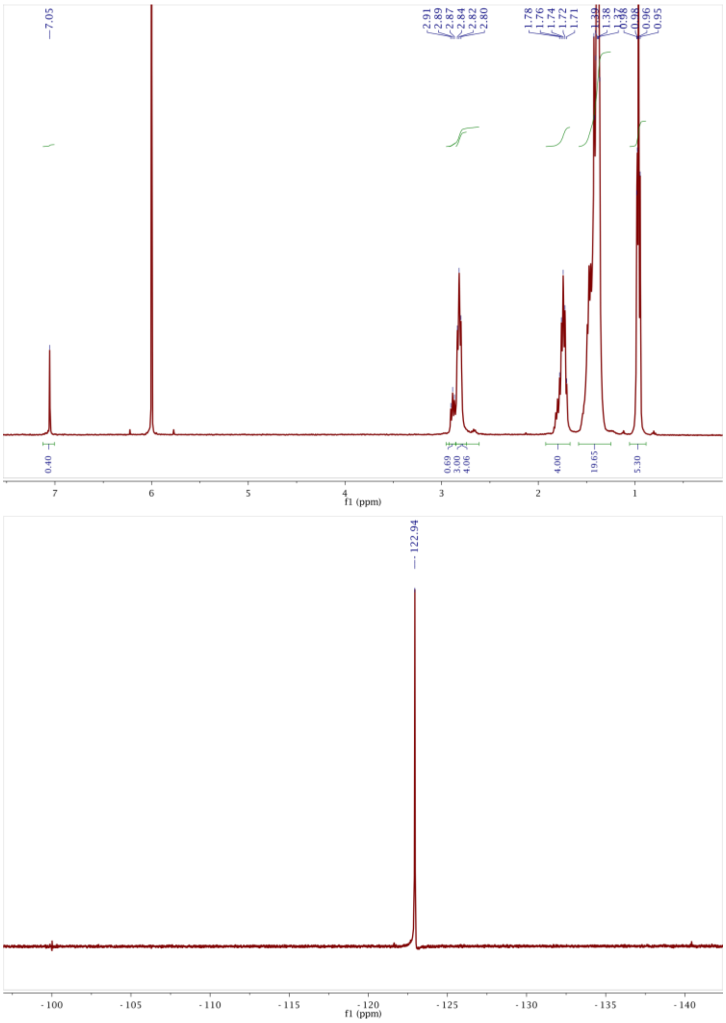

Figure S2: NMR P3OT-*b*-F-P3OT 1:4.

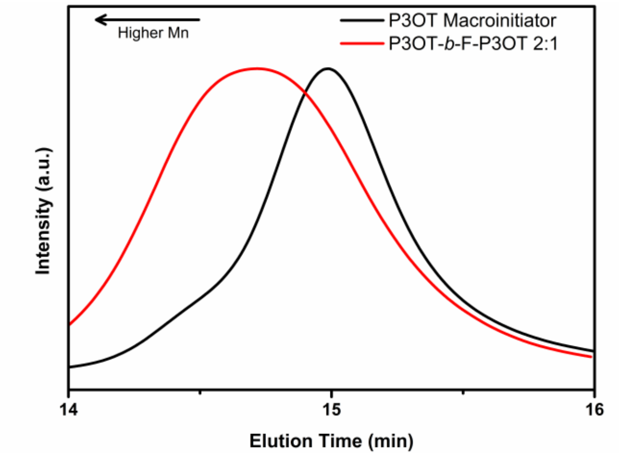

Figure S3: Gel Permeation Chromatography trace during the synthesis of P3OT-*b*-F-P3OT 2:1.

The black trace is the P3OT macroinitiator sample taken prior to the addition of 4, and the red trace is P3OT-*b*-F-P3OT 2:1 after Soxhlet purification.

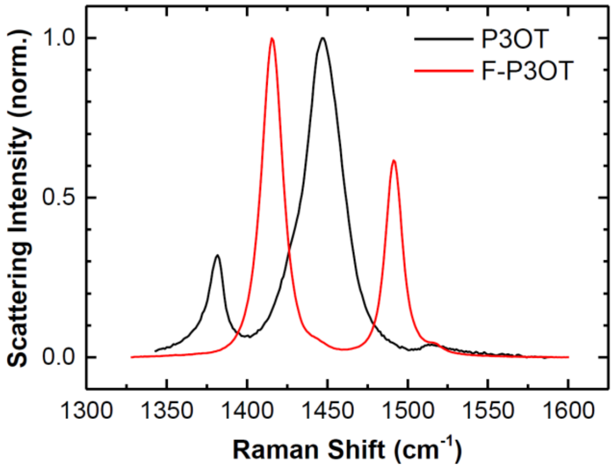

Figure S4: Room temperature Raman spectra of P3OT and F-P3OT homopolymers.

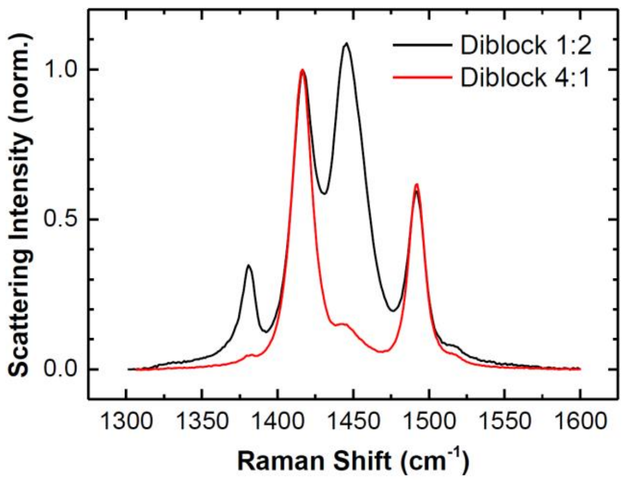

Figure S5: Room temperature Raman spectra of P3OT-*b*-F-P3OT 2:1 and P3OT-*b*-F-P3OT 1:4.

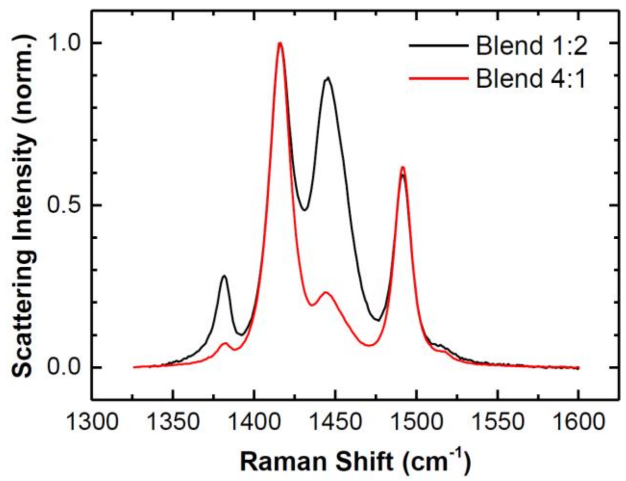

Figure S6: Room temperature Raman spectra of blends of P3OT and F-P3OT in 2:1 and 1:4 ratios.

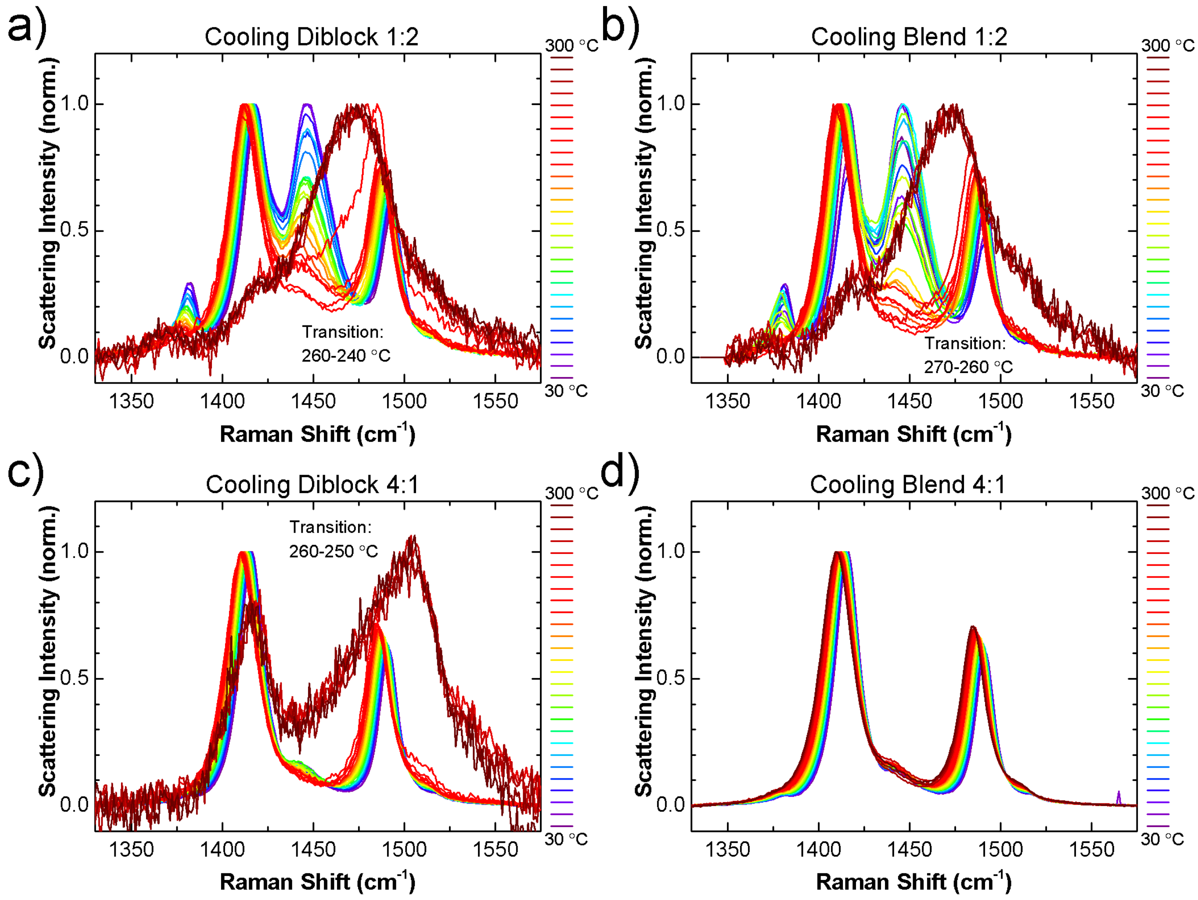

Figure S7: Temperature dependent Raman spectra measured during cooling thin film samples from 300 °C to 30 °C, (*cf.* heating data in Figure 6) showing the main C-C and C=C stretches of a) P3OT-*b*-F-P3OT 2:1, b) P3OT/F-P3OT blend in a 2:1 ratio, c) P3OT-*b*-F-P3OT 4:1, and d) P3OT/F-P3OT blend in a 4:1 ratio.
